# IBX-Mediated Organic Transformations in Heterocyclic Chemistry-A Decade Update

**DOI:** 10.3389/fchem.2022.841751

**Published:** 2022-02-28

**Authors:** Yadavalli Venkata Durga Nageswar, Katla Ramesh, Katla Rakhi

**Affiliations:** ^1^ Retired Chief Scientist, Indian Institute of Chemical Technology-IICT, Hyderabad, India; ^2^ Organic Chemistry Laboratory, Foreign Visiting Professor, School of Chemistry and Food, Federal University of Rio Grande-FURG, Rio Grande, Brazil; ^3^ Organic Catalysis and Biocatalysis Laboratory-LACOB, Federal University of Grande Dourados-UFGD, Dourados, Brazil

**Keywords:** 2-iodoxybenzoic acid (IBX), multicomponent-reactions, heterocyclic compounds, aza-Friedel–Craft reactions, Ugi-type three-component reaction

## Abstract

O-Iodoxybenzoic acid (IBX) is a very mild and efficient hypervalent iodine synthetic reagent useful to carry out several selective oxidations. The present review highlights research reports on IBX-assisted transformations in heterocyclic derivatives, particularly from 2010 onward.

## Introduction

Hypervalent iodine chemistry evolved into an important area of research and application after the first discovery of the hypervalent iodine reagent (PhICl_2_) iodobenzene dichloride in 1886 (Willgerodt, 1886). These hypervalent iodine (III) compounds represent attractive oxidizing agents due to their selective actions and stability. Iodosyl benzene (PhIO) iodobenzene dichloride (PhICl_2_), iodobenzene diacetate (PhI(OAc)_2_), iodine trifluoroacetate [(PhI(OCOCF_3_)]_2_, Koser’s reagent-hydroxy (tosyloxy) iodobenzene (PhI(OTS)OH) (HTIB), Dess–Martin periodinane (DMP), and 2-iodoxybenzoic acid (IBX) are the widely used hypervalent iodine reagents for organic transformations (Dong et al., 2014; Sun and Shi, 2014; Charpentier et al., 2015; Li et al., 2016; Yoshimura and Zhdankin, 2016; Hyatt et al., 2019), due to the mild reaction conditions and transition metal-free nature and operational simplicity. These are popularly employed for C–H functionalization (Kandimalla et al., 2019), bifunctionalization of olefins and cyclopropane (Banik et al., 2017; Muñiz et al., 2017), and oxidative cyclizations (Chi et al., 2014; Xing et al., 2019; Zhen et al., 2019). Several articles appeared in recent literature highlighting the significance of these reagents in modern organic synthesis and in particular catalytic strategies. These are proved to be unique in conducting many important rearrangement reactions providing useful building blocks.

2-Iodoxybenzoic acid (IBX) is the most important representative of pentavalent iodine compounds, apart from DMP (Hartman and Mayer, 1983). Despite having drawbacks such as explosive nature and insolubility in common organic solvents, IBX is widely applied for organic oxidative reactions, particularly for the selective oxidation of alcohols to carbonyl compounds, even in complex structural entities with acceptable functional group tolerance. IBX can be easily prepared by the oxidation of 2-iodobenzoic acid. Many reactions with IBX are performed in DMSO, due to solubility. When solvents such as DCM, DCE, ACN, and EtOAc are used, the reactions may need higher temperatures.

Heterocyclic compounds widely occur in nature and play a significant role in human life. A vast number of these are pharmacologically active. These moieties represent different and divergent classes of molecules such as drugs, amino acids, and dye stuffs; a large number of heterocyclic compounds are known in the literature and are being synthesized regularly.

The present review highlights research reports on IBX-assisted transformations in heterocyclic derivatives, particularly from 2010 onward.

## Applications of IBX in the Synthesis of Heterocycles

In an interesting research article submitted by Jorg. T. Binder and Stefan F. Kirsch, tetra- and penta-substituted 5-methyl pyrroles 1) were conveniently prepared *via* a multimetal-catalyzed rearrangement condensation–cyclization domino approach by a one-pot process, utilizing easily available aromatic amines 2) and propargyl vinyl ethers 3) as reactants (Supplementary Figure S1) (Binder and Kirsch, 2006).

In the first step of this cascade process, propargyl vinyl ethers 3) formed corresponding allenyl carbonyl compounds in the presence of low catalyst loadings of AgSbF_6_ at 23°C, without any significant by-product formation. In the second step, these intermediary compounds undergo condensation with aryl/heteroaryl amines 2) followed by cyclization resulting in the highly substituted pyrrole derivatives 1) in the CH_2_Cl_2_ medium. In the last stage of the process, 5-exo-dig-hetero cyclization was facilitated by (Ph_3_P) AuCl (5 mol%) at 38°C. Aliphatic amines 2a) did not provide the corresponding products. A broad range of propargyl vinyl ethers 3) was used effectively. Even though, the approach was favorable for the formation of 5-methyl-pyrrole-3-carboxylates 1), the methyl group is amenable for alkylation–halogenation and Mannich reactions. Interestingly, the authors employed IBX to obtain valuable 5-formyl pyrroles 4) in the presence of DMSO at 110°C (Supplementary Figures S2,S3).

Pravin Patil et al. developed direct oxidative arylation of naphthoquinones 8) employing *o*-iodoxybenzoic acid and aryl hydrazines 9) in acetonitrile at room temperature (Supplementary Figures S4,S5) (Patil et al., 2014). Authors screened various hypervalent iodine (V) reagents such as iodic acid (HIO_3_), Dess–Martin periodinane (DMP), and IBX for their efficiency. It was observed that DMP and HIO_3_ afforded 67 and 56%, respectively, when 2-amino-1,4-naphthoquinone was used as a standard material, whereas IBX gave 78% of the desired product. THF, CHCl_3_, toluene, ACN, and DMSO were tried as solvents, and out of these ACN was found to be ideal. DMSO yielded multiple side products. The best results observed that 2.0 equivalents of IBX and 1.2 equivalents of phenylhydrazine were used. Substrate scope was studied using several substituted phenylhydrazines 9), and 2-substituted naphthoquinones 8) were used. Authors proposed that the reaction proceeded through the intermediary of an aryl radical generated through the oxidation of the aryl hydrazine 9) molecule by IBX.

The protocol has been applied for the synthesis of the antitumor and antibiotic precursor benzocarbazoledione 11).

Nishanth Verma and co-authors employed LiBr/β-CD in the H_2_O–DMSO mixture for highly regioselective ring cleavage of epoxides 12) affording bromohydrins 13) (Supplementary Figure S6). These bromohydrins 13) were later converted successfully into 1, 2, 3-triketones 14) by the IBX-mediated oxidation in DMSO under room temperature conditions (Verma et al., 2017). Authors reported moderate to good yields for these products.

Narendar Reddy Gade et al. described the synthesis of highly functionalized pyridines 15) by a one-pot metal-free domino approach (Gade et al., 2013). Authors designed this interesting domino reaction between *ß*-enamino esters 16) and allyl alcohols 17) utilizing 2-iodoxybenzoic acid as an oxidant and carried out reactions in the DMSO medium (Supplementary Figures S7,S8). It was observed that the reaction did not proceed in the absence of IBX and also in the presence of other oxidants such as Ag_2_O. A broad range of 2-substituted nicotinic acid derivatives was made by widening the substrate scope to aryl/heteroaryl/benzyl/cinnamoyl/aliphatic *ß*-enamino esters 16a). The approach was also extended to include various allylic alcohols 17). However, 4-substituted pyridines could not be prepared by this methodology.

This approach was also used to prepare the pyridine core of cyclothiazomycin 18) (Supplementary Figure S8).

A green approach for the synthesis of a library of 3,4-dihydro pyrimidine-2(1*H*)-ones 21) (DHPMS) in the water medium, *via* the Biginelli reaction was developed by Santosh Takale and co-authors employing several aromatic aldehydes (22), *ß*-keto esters 23) such as methyl/ethyl acetoacetate, and thiourea/urea 24) as reactants in the presence of iodoxybenzoic acid at 60°C (Supplementary Figure S9) (Takale et al., 2011). In the initial studies, authors screened several catalysts such as TBHS, *ß*-CD, CuCl, CAS, iodine, DABCO, and DHP apart from IBX for their efficacy and observed that IBX afforded the best results for the reaction. Studies were also conducted to select a better solvent for which water, methanol, ethanol, chloroform, dichloromethane, DMF, acetonitrile, THF, and DMSO were examined. The reactions carried out under solvent-free conditions and water medium provided better results. The protocol was compatible with different functional groups, including nitro, cyano, hydroxy, methoxy, and halide groups. It was reported that 60% of IBX was recovered and reused without loss of activity for some of the following reaction cycles.

C. de Graaff et al. unveiled that IBX promoted mild and selective oxidation of unactivated cyclic amines (2b) to imines (25), employing a range of aliphatic meso-pyrrolidines (2b) as substrates (Supplementary Figure S10) (de Graaff et al., 2015) The protocol was further applied for diastereoselective oxidative Ugi-type and aza-Friedel–Crafts reactions (26, 27) (Supplementary Figures S11,S12). Authors assessed the efficacy of different commercially available hypervalent iodine reagents, such as (diacetoxy) iodobenzene (PIDA), Dess–Martin periodinane (DMP), [bis(trifluroacetoxy)]iodo benzene (PIFA), and *o*-iodoxybenzoic acid (IBX). Over-oxidation was not observed in case of bi- and tri-cyclic-1-pyrrolines. Authors successfully employed various aliphatic and aromatic isocyanides and electronically diverse carboxylic acids as compatible reactions in the Ugi-type reactions. In the one-pot two-step aza-Friedel–Crafts reaction, 2-substituted pyrrolidines 27) were obtained from suitable nucleophiles in the presence of TFA, in modest to good yields and high to excellent diastereoselectivity.

An efficient mild one-pot sequential multi-component approach for providing an easy access to N-aryl-pyrrole-3-carbaldehydes 28) under metal-free conditions was accomplished by (Supplementary Figures S13,S14) Singh et al. (2018). The protocol includes a proline-catalyzed direct Mannich reaction–cyclization sequence between the *in situ*-generated aryl/heteroaryl/indolyl-imines and succinaldehyde (29). It is followed by IBX-supported oxidative aromatization affording the title compounds. The protocol was efficiently applied for generating diverse bioactive fused heterocyclic scaffolds such as pyrrolo-oxadiazole, dihydro pyrrolo-quinoline, pyrrolo-quinoline, and pyrrolophenanthridine.

Initially, authors examined the effect of solvents such as DMF, CH_3_CN, and DMSO and oxidants such as K_2_S_2_O_8_, oxone, and IBX. The best results for oxidative aromatization are provided with DMSO as a solvent and IBX as an oxidant at 70°C, in 4 h duration.

The scope of the reaction was extended to include aromatic aldehydes 22) decorated with different functional groups such as F, Cl, Br, CN, NO_2_, and CF_3_ at o-, m-, and p-positions as well as various substituted indole-3-aldehydes (24a).

Zsofia Makra et al. established a convenient metal-free, IBX–NIS-assisted intramolecular oxidative annulation of Mannich-type substrates affording imidazol [1,2-*a*] fused bicyclic 32) frame works such as imidazol [1,2-*a*]pyridine (33), imidazo [1,2-*a*]pyrimidine 34) and imidazo [1,2-*a*]pyrazines 35) in excellent yields (Supplementary Figures S15–S17) (Makra et al., 2019). The present industrially compatible, eco-friendly methodology includes iodination, NH-oxidation, intramolecular C–N-bond formation, and retro-Claisen–Schmidt sequence. It is also useful for utilizing highly diverse Mannich substrates/intermediates for oxidative C (sp^3^)-functionalization, with a further derivatization potential.

Initially, Mannich precursors were prepared by the reaction of primary aromatic amines (2), *ß*-keto esters 23), and aryl/alkyl/pyridyl aldehydes 24) in the presence of phosphotungstic acid (PTA) in H_2_O/MeCN. The Mannich precursors were subjected to intramolecular oxidative annulation in DMA at 80°C in good to excellent yields affording imidazo [1,2-*a*] fused bicyclic scaffolds promoted by the oxidant IBX and additive NIS. During optimization studies, the authors examined the efficacy of the solvents such as MeCN/C_6_H_6_/DCE/EtOH/tBuOH/THF/DMF/DMA/DMSO/toluene. Various oxidants, such as PIFA, DMP, PIDA, and IBX were screened. Additives such as NIS, NBS, NCS, I_2_, and IPT were examined for achieving best results. Authors produced a highly diverse 30-membered libraries of imidazo [1,2-*a*]pyrimidines 37) following the standardized procedure (Supplementary Figure S16). Following the same synthetic strategy, Mannich substrates related to pyridine 38) and pyrazine ring systems were also investigated successfully to access highly diverse cyclic compounds imidazo [1,2-*a*]pyridines (39) and imidazo [1,2-*a*]pyrazines (40) with moderate to good yields (Supplementary Figure S17).

An interesting mild one-pot two-step metal-free methodology for synthesizing tetracyclic oxazepine-fused pyrrole scaffolds 42) was explored by Sachin Choudhary and co-researchers through [3 + 2] the annulation strategy between dibenzo [b,f][1,4] oxazepines 43) and aqueous succinaldehyde 29) involving proline-catalyzed direct Mannich/cyclization followed by the IBX-supported oxidation sequence (Supplementary Figure S18) (Choudhary et al., 2019). Among solvents, DMSO was proved to be useful. For the oxidation step, authors examined the efficacy of several oxidants, such as SeO_2_, K_2_S_2_O_8_, DDQ, oxone, and IBX. Authors claim that the high solubility of IBX in DMSO and its ability to dehydrogenase carbonyl functionality with the corresponding α, *ß*-unsaturated moieties made this protocol feasible. A broad range of 1,4-dibenzoxazepines 43) decorated with a variety of electron-withdrawing and electron-donating functionalities in both rings was exploited as convenient substrates for the protocol.

Authors explained the reaction mechanism. Authors also further investigated on possible further functionalization of the scaffolds by feasible transformations.

Several antioxidants and anti-influenza moieties containing pyrogallol 44) and catechol structures were made by Bruno. M. Bizzarri et al. from coumarin 45) by the oxidation employing 2-iodoxybenzoic acid in DMSO (Supplementary Figures S19,S20) (Bizzari et al., 2017). High regioselectivity was observed in the product formation. Authors also attempted on large-scale synthetic applications employing readily recoverable IBX-supported on polystyrene (PSS-IBX). The reaction mechanism for the product formations was explained appropriately.

An interesting rapid and efficient protocol was presented by Alejandro Islas-Jacome and co-authors for a series of four novel nuevamine aza analogs containing the isoindolo [1,2-*a*] isoquinoline 56) skeleton (Supplementary Figure S21) (González-Zamora et al., 2014). This high-yielding approach involves IBX-promoted oxidation of secondary amine (2b) followed by isonitrile-α-addition and aza-Diels–Alder cycloaddition. Authors used combinedly an oxidative Ugi-3CR with an aza-Diels–Alder cycloaddition as a post-condensation process. The sequence of steps starts with IBX oxidation of 1,2,3,4-tetrahydro isoquinoline 57) in refluxing THF affording 3,4-dihydroisoquinoline iminium ion followed by the addition of *a*-isocyano-α-benzyl acetamides (58). These adducts undergo ring chain tautomerization yielding 5-amino oxazole derivatives, which on intermolecular acylation followed by intramolecular aza-Diels–Alder cycloaddition producing oxa-bridged intermediates. Title compounds are obtained by the dehydration and decarboxylation from these intermediates in excellent yields.

The methodology presents formation of six new chemical bonds and four fused rings from readily available reactants in three simple synthetic processes in the one-pot operation as claimed by the authors.

Dalip Kumar et al. presented an expeditious one-pot-approach for *a*-keto-1,3,4-oxadiazoles 61) mediated by IBX/tetraethyl ammonium bromide in an oxidative cyclization of hydrazide–hydrazones (9c), which were produced *in situ* from the reaction of hydrazides (9b) and aryl glyoxals 62) under mild conditions (Supplementary Figures S22,S23) (Kumar et al., 2014). Authors also prepared *a*-keto-1,2,4-triazolo [4,3-a]pyridines 63) from aryl glyoxals (62a) and 2-hydrazino pyridine (9d) mediated by IBX/TEAB. After screening temperatures and stoichiometries, authors employed IBX (1 equiv) and TEAB (1.2 equiv) for the oxidative cyclization.

Highly functionalized indolizine scaffolds 64) were produced by Thiago. S. Silva and co-authors by a fast, mild, atom-efficient protocol involving sequential one-pot IBX oxidation of Morita–Baylis–Hillman (MBH) adducts and catalyst-free conjugate addition of indolizine derivatives 65) as nucleophiles (Supplementary Figure S24) (Silva et al., 2020). The reactions proceeded with high regioselectivity with regard to C-3 position of indolizines (67), since C-1 position was less nucleophilic than C-3. During optimization studies, authors examined the feasibility and effects of different temperature conditions and stoichiometries. Post-optimization, scope of MBH adducts, and indolizine 64) substitutions were extensively evaluated and explored by the authors.

Authors expanded the studies to include MBH adducts derived from acrylamide, t-butyl acrylate, and vinyl oxadiazoles, producing newer heterocyclic assemblies. Authors also investigated on relative nucleophilicity of indolizines/indoles 67) and concluded that the indolizine skeleton 67) appeared to be a superior nucleophile in the present reaction conditions.

2-Aryl benzoxazoles 68) were obtained by a simple protocol by the IBX oxidation of phenolic Schiffs’ bases (2d) in the presence of 4A^o^ molecular sieves as described by Fei Chen et al. in their research article (Supplementary Figures S25,S26) (Chen et al., 2011).

The methodology was extended to prepare some arylbenzoxazoles containing amino acids 69).

Kamlesh Kumar et al. developed an efficient and practical (±)-camphor sulfonic acid-aided IBX-supported oxidation of a broad range of primary and secondary alcohols (70) belonging to aliphatic/aromatic/heterocyclic systems decorated with differently substituted functionalities (Supplementary Figure S27) (Kumar et al., 2021). The role of the CSA/IBX catalytic system in the reaction was presented mechanistically.

Matrine-type alkaloids (72), having broad and potent biological activities, are widely present in the *Sophora flavescens*, *S. alopecuroides*, and *S. subprostrara*. Out of the total content of alkaloids of Sophora alopecuroides, 6% accounts for sophocarpine (73), which is convenient for derivatization due to the presence of conjugated double bond as an α, *ß*-unsaturated lactam system. Chaojie Li and co-authors in an interesting research communication described a metal-free, eco-friendly, and operationally simple approach for the semisynthesis of sophocarpine 73) from matrine 72) in 91% yield, *via* the formation of 14-phenyl sulfinyl matrine 74) with efficient and controllable oxidation of thioether 74) to sulfoxide 75) by IBX/H_2_O as the key step (Supplementary Figures S28,S29) (Li et al., 2014). During study, authors examined the feasibility of several bases and solvents such as NaH/THF, NaH/PhCH_3_, LDA/THF, and LiHMDS/THF for the direct sulfinylation of matrine 72) using methyl phenyl sulfinate with little or no desired product formation. Finally, authors succeeded by reacting matrine 72) with diphenyl disulfide in the presence of LDA to obtain 14-thio phenyl matrine 74) in 96% yield followed by IBX oxidation in acidic aqueous solution in 95% yield.

A large library of aryl/heteroaryl trifluoromethyl alcohols 77) and aryl/heteroaryl trifluromethyl ketones 78) was prepared by Huicheng Cheng and co-authors from the corresponding aldehydes 22) in excellent yields (Supplementary Figures S30,S31) (Cheng et al., 2013). Initially, TMS-protected trifluoromethylated alcohols were obtained from the respective aldehydes 22) by reacting with TMSCF_3_ 79) in the presence of the catalytic amount of K_2_CO_3_ in DMF at room temperature. For the oxidation step, authors screened oxidants such as DMP, PCC, and Swern apart from IBX. Two equivalents of IBX in EtOAc under reflux conditions provided ketones 80).

IBX was observed to be a better effective reagent to both electron-rich and electron-deficient benzyl alcohols 77). For heteroaryl alcohols (71a), IBX was found to be suitable to pyridines, indoles, furans, benzofuranes, thiophene, and benzothiophene systems. The process allows recycling and reuse of IBX and found to be scalable.

Zhinguo Zhang and co-researchers investigated on the IBX-mediated intramolecular cascade cyclization reaction of tryptophan analogs bearing the N-aryl amides 81) side chain resulting in the formation of polycyclic spiro-fused indoline scaffolds 82), with multiple stereocenters including quaternary stereocenters (Supplementary Figure S32) (Zhang et al., 2020). Authors claim that this tandem spiro-fused cyclization exhibits highly efficient construction of two stereocenters in one step, including one quaternary carbon stereocenter containing five to eight rings, aiming at structurally diverse oxazine-containing complex polycyclic indolines.

Authors evaluated the scope of various tryptophan analogs 81). N-protecting groups were well tolerated. Various amino groups on the side chain, such as NHCbZ, NHBoc, and N_3_ were tolerated, and N-unprotected substrate did not give the product. Authors observed that the substitution on the quinoline ring strongly influenced the reaction. Methyl and bromo groups on the indole moiety also gave expected products. Authors explained the mechanism of this interesting and important cascade reaction.

Cheng-Kun Lin and Ta-Jung Lu reported a simple and practical approach for the IBX-assisted oxidation of primary aliphatic (77), benzylic (77a), and allylic alcohols, in the presence of stoichiometric quantity of acetic acid. After initial studies, authors observed that the reaction proceeded well in the presence of 1.2 eq. of IBX and 1.2 eq. of AcOH in acetonitrile solvent (Supplementary Figure S33) (Lin and Lu, 2010). All the primary alcohols 71) were oxidized to aldehydes 22) in good yields (90–97%). The reaction time changed significantly depending on the substituent pattern on the benzene ring of benzyl alcohols (77a). An important observation is that the addition of AcOH in other solvent systems did not show improved yields and conditions. It was presumed that AcOH acted as an external proton source in assisting and liberating the water molecule from the intermediate and might be accelerating the oxidation.

In an interesting research article, Maurizio Barontini and co-researcher brought out selective and effective oxidative modification of flavonoids employing 2-iodoxybenzoic acid as well as IBX-polystyrene producing a large panel of biologically active compounds (Supplementary Figures S34,S35) (Barontini et al., 2010). Authors examined the efficiency of IBX in the oxidation of a series of flavanones 84) and flavones 90).

In either case, a hydroxyl group on C-5, due to hydrogen bond formation with oxygen of the carbonyl group on C-4 hindered the formation of the *λ* ^5^-5-iodanyl complex with IBX, making both 5-hydroxy flavone 94) and 5-hydroxy flavanone 88) unreactive irrespective of reaction temperatures. Excepting these two compounds, other hydroxy flavones 90) and flavanones 84) produced aromatic o-hydroxylated products in both the series. Authors also successfully examined the efficiency and selectivity of recyclable IBX-polystyrene toward the dehydrogenation reaction of methoxylated flavanones 86). Over all, the authors reported regioselective aromatic hydroxylation using IBX (1.2 eq) under room temperature conditions resulting in anti-oxidant dihydroxylated flavonoids and dehydrogenation of selected methoxy flavanones 96) to anticancer flavones 9**9**) employing IBX-polystyrene.

Kamlesh Kumar et al. described an efficient scalable and practical method for the IBX–TfOH-assisted oxidation of primary and secondary alcohols 77) in 1,4-dioxane at ambient temperature to afford aldehydes 22) and ketones (103) in quantitative yields (Supplementary Figure S36) (Kumar et al., 2020). The protocol covers a broad range of substrates with alkene, arene, and heteroarene functionalities. It was reported that the title compounds are formed without the formation of over-oxidation products. During the optimization studies, different solvents and stoichiometric ratios are examined. A plausible mechanism for the acid-catalyzed oxidation of alcohols with IBX was explained.

Girish Prabhu and V. V. Sureshbabu presented a mild and rapid simple process for 2-amino-1,3,4-oxadiazoles 104) obtained by the IBX-mediated cyclo-desulfurization of intermediate acylthiosemicarbazides starting from the corresponding acyl hydrazides 9b) (Supplementary Figure S37) (Prabhu and Sureshbabu, 2012).

During optimization, different solvents such as DMF, MeCN, EtOAc, and THF were screened for the efficacy. The stoichiometry of IBX and TEA was fixed after studies. The protocol covered a variety of acyl hydrazides (9b) and isothiocyanates (105). A mechanism was proposed for the IBX-supported cyclo-desulfurization of acylthiosemicarbazides. The method was also extended to cyclo-deselenization of acylselenosemicarbazides, confirming that IBX acted effectively for both cyclo-desulfurization and cyclo-deselenization affording the title compounds in good to excellent yields.

A simple mild metal-free two-step approach was developed for 2-amino benzoxazole derivatives (106) by Yogesh S. Wagh et al. *via* C–H bond amination of benzoxazoles 107) using amines 2) through the ring-opening/ring-closure method (Supplementary Figure S38) (Wagh et al., 2013). It involves catalyst-free nucleophilic addition of amines 2) on benzoxazoles (107) under solvent-free conditions, followed by IBX-assisted oxidative ring closure. The protocol is useful for cyclic, acyclic, and functionalized aliphatic amines (2). Authors examined the efficacy of KIO_3_, KIO_4_, HIO_4_, H_2_O_2_, O_2_, and IBX for the oxidative ring-closure step. CH_2_Cl_2_ was observed to give the best results as compared to other solvents such as CHCl_3_, CH_3_CN, THF, toluene, and DMSO.

Narayana Murthy and Nageswar explored *o*-iodoxybenzoic acid as an efficient and mild oxidizing agent for aromatizing diversely substituted 1-benzylpyrrolidines (108) and *N*-substituted *l* -proline analogs (110) in the aqueous medium assisted by the supramolecular host *ß*-CD under room temperature conditions (Supplementary Figures S39,S40) (Murthy and Nageswar, 2011). Authors noticed that in the absence of *ß*-CD, these reactions did not result in satisfactory yields of the aromatized products. The scope of the study was enlarged to include various diversely substituted *N*-benzyl pyrrolidines 108) and a wide variety of *N*-protected *l* -proline esters.

The synthesis of quinazolines 111) and dihydroquinazolines 112) from commercially available starting materials including aliphatic (22), aromatic, and heteroaromatic aldehydes (22, 22a) and *o*-aminobenzylamine (2d) was achieved by the authors Subhabrata Sen and Santanu Hati (Supplementary Figure S41). They employed IBX as a metal-free oxidant in acetonitrile at ambient temperatures. (Sen and Hati, 2016). Authors synthesized a total of 33 compounds using 1 equiv. or 2 equiv. of IBX. The desired products were obtained in the range of 50–96%.

Several oxidants including IBX, NBS, urea-hydrogen peroxide (UHP), *tert-*butyl hydroperoxide, *m*-chloroperoxybenzoic acid, and benzoyl peroxide were evaluated in the optimization process. Other oxidants, with the exception of IBX, resulted in lower yields of the desired products.

During optimization studies, the effect of polar aprotic, polar protic, and nonpolar solvents was also examined. A polar aprotic solvent such as acetonitrile provided high yields of the desired compound. In addition, aryl (22), heteroaryl (22a), and alkyl aldehydes (22c) interacted well with their coupling partners.

2-Iodoxybenzoic acid was conveniently utilized by Joseph D. Panarese and Stephen P. Waters for aromatization of tetra hydro-β-carbolines under mild conditions, in acetonitrile at room temperature in the presence of TBAB (Panarese and Waters, 2010). The method was applied by the authors in the four-step total synthesis of the marine alkaloid eudistomin U (115), with an indole skeleton 113) (Supplementary Figure S42). It was observed that the presence of the ester group at C 3) appeared to be important for successful aromatization. The electron-donating groups at C 1) afforded higher yields. The aromatic functional groups were well tolerated, and indole 113) nitrogen need not be protected for the reactions to proceed fruitfully under the present reaction conditions. The aliphatic and heteroaromatic substituents at C 1) also did not hinder the reaction.

A mild room temperature oxidative dimerization of thioamides 116) under the influence of the IBX/TEAB system in acetonitrile solvent to afford 3,5-disubstituted-1,2,4-thiadiazoles 117) in high yields was described by (Supplementary Figure S43) Patil et al. (2009). Authors carried out these reactions successfully involving substrates from aromatic (118), heteroaromatic, and benzylic thioamides.

## Conclusion

Among the metal-free oxidants, IBX occupies a lead position with its vast applications in synthetic organic chemistry including many total syntheses. IBX-mediated transformations are mild and eco-friendly. Further scope of the applications of IBX may widen in coming years, with more preparations of soluble as well as recyclable modifications of IBX coming out and refinement of working methodologies.

The authors of this review acknowledge the original contributors and publishers of the articles cited here. Researchers are advised to refer original articles for any detailed information. All the figures are redrawn to give an idea about the research work discussed in the text.
